# Hypothesis testing in Bayesian network meta-analysis

**DOI:** 10.1186/s12874-018-0574-y

**Published:** 2018-11-12

**Authors:** Lorenz Uhlmann, Katrin Jensen, Meinhard Kieser

**Affiliations:** Institute of Medical Biometry and Informatics, University of Heidelberg, Im Neuenheimer Feld 130.3, Heidelberg, Germany

**Keywords:** Network meta-analysis, Hypothesis testing, Treatment comparison, Superiority, Non-inferiority

## Abstract

**Background:**

Network meta-analysis is an extension of the classical pairwise meta-analysis and allows to compare multiple interventions based on both head-to-head comparisons within trials and indirect comparisons across trials. Bayesian or frequentist models are applied to obtain effect estimates with credible or confidence intervals. Furthermore, p-values or similar measures may be helpful for the comparison of the included arms but related methods are not yet addressed in the literature. In this article, we discuss how hypothesis testing can be done in a Bayesian network meta-analysis.

**Methods:**

An index is presented and discussed in a Bayesian modeling framework. Simulation studies were performed to evaluate the characteristics of this index. The approach is illustrated by a real data example.

**Results:**

The simulation studies revealed that the type I error rate is controlled. The approach can be applied in a superiority as well as in a non-inferiority setting.

**Conclusions:**

Test decisions can be based on the proposed index. The index may be a valuable complement to the commonly reported results of network meta-analyses. The method is easy to apply and of no (noticeable) additional computational cost.

## Background

Network meta-analysis (NMA), as an extension of the classical pairwise meta-analysis, is gaining acceptance and popularity in medical research. The general idea is to include all evidence at hand about a specific research question in one single model. The classical pair-wise meta-analysis is limited to two-arm comparisons of interventions that were directly compared in trials. An NMA can include any number of treatments as well as interventions that have not been investigated head-to-head. Several approaches (frequentist and Bayesian) were introduced and extended during recent years. Thus, a framework of modeling techniques is available to implement an NMA in many different data situations. Efthimiou et al. and Dias et al. give very useful overview of recent developments [1, 2]. Alongside the benefits those procedures provide, many challenges arise when applying an NMA model. First, all the issues that are already known from pair-wise meta-analysis, like heterogeneity, have to be addressed. In addition, new items, like inconsistency which denotes the problem of deviations between direct and indirect estimates, have to be taken into consideration (see, for example, Dias et al. [3]).

As a result of an NMA, point estimates with credible intervals of pairwise effects between treatment arms are obtained. In this article, we focus on the issue of testing for superiority or noninferiority between treatment arms in an NMA model. For Bayesian modelling, we present and discuss an index *υ* that can be used for hypothesis testing within the network. Similar ideas were presented in the article by Rücker and Schwarzer [4] in a frequentist framework. However, we focus on Bayesian modeling. Furthermore, while we apply the index for a test procedure, Rücker and Schwarzer use their approach to rank treatment arms.

### General modeling in NMA

The concept of NMA in a Bayesian framework was introduced by Higgins and Whitehead [5]. Many extensions and discussions about the idea were published in recent years. Introductions and overviews can be found in the literature [1, 2, 6, 7]. Here, we only present the basic idea of the modeling procedure. For this, we assume throughout this paper that the outcome is binary (e.g., success / no success, or failure / no failure).

The following notation is used. *N* is the number of trials, *K* the number of arms, *p*_*ik*_ the success (or failure) probability, and *N*_*ik*_ the sample size of arm *k* in study *i*. In the setting of a binary outcome, we apply two different approaches: Either by use of the binomial distributions directly or by calculating the log odds ratios (OR) for each trial which are pooled in the model afterwards. In the former case, we use the logit function as link function and assume

1 $$ \begin{aligned} y_{ik} &\sim \text{Bin}(N_{ik}, p_{ik}),\\ \text{logit}(p_{ik}) &= \mu_{i} + d_{A_{i}k}, \end{aligned}  $$

which can be denoted as a fixed-effect model, where *y*_*ik*_ is the number of events, *μ*_*i*_ is the baseline value (and is seen as a nuisance parameter), $d_{A_{i}k}$ is the log OR between arm *k* and arm *A*_*i*_ which is the baseline arm and has to be chosen for each trial. All arms are compared to this baseline treatment arm. These log ORs are of main interest in an NMA and are typically assumed to be approximately normally distributed. In a random-effects model, the logits are modeled as

$$\begin{array}{*{20}l} \text{logit}(p_{ik}) &= \mu_{i} + \delta_{A_{i}k}\\ \delta_{A_{i}k} &= \mathcal{N}(d_{A_{i}k}, \tau^{2}). \end{array} $$

When the log ORs are used directly, the fixed-effect model is defined as

2 $$ \psi_{{iA}_{i}k} \sim \mathcal{N}(d_{A_{i}k}, \text{var}(\psi_{{iA}_{i}k}))   $$

and a random-effects model as

$$\begin{array}{*{20}l} \psi_{{iA}_{i}k} &\sim \mathcal{N}(\delta_{A_{i}k}, \text{var}(\psi_{{iA}_{i}k}))\\ \delta_{A_{i}k} &= \mathcal{N}(d_{A_{i}k}, \tau^{2}). \end{array} $$

In this implementation, $\phantom {\dot {i}\!}\psi _{{iA}_{i}k}$ is the log OR in trial *i* of treatment arm *k* compared to the baseline treatment arm *A*_*i*_. The log OR together with its variance $\phantom {\dot {i}\!}\text {var}(\psi _{{iA}_{i}k})$ have to be estimated using the data of study *i*. The estimation of $\phantom {\dot {i}\!}\psi _{{iA}_{i}k}$ can be problematic when the number of events is rare (see [8–10], and the Cochrane Handbook, chapter 16.9.2 [11]). Thus, some care has to be taken when applying this approach. Further challenges and assumptions (as, for instance, the consistency assumption) but also extensions of these models are discussed and explained in the literature. Albeit there are important issues, we do not focus on them here.

### Objective

In this paper, we want to introduce a simple method to obtain an index *υ* that can be interpreted similarly to a frequentist p-value for an effect estimate within a Bayesian NMA. For this, we adapt an idea proposed by Kawasaki and Miyaoka [12, 13] where the authors introduce a similar index but to compare only two groups with respect to a binary outcome using Bayesian methods in a randomized trial. Our approach serves as a complement when presenting the results of an NMA reporting the effect estimates and the credible intervals. It can also be interpreted as the probability of superiority or non-inferiority, respectively. Furthermore, the index might be useful to define boundaries when updating NMAs as proposed by Nikolakopoulou et al. [14] and may therefore be applied in sequential NMAs. In our simulation study and real data example, we discuss the characteristics of the proposed approach.

## Methods

In this section, we present the definition of the index *υ* and how it can be used when comparing two treatment arms within an NMA model.

### Definition of index *υ*

To explain our approach, we assume that there are three treatment arms compared (*P*: Placebo, *S*: standard treatment, and *E*: experimental treatment). Assuming that an event denotes a success, a log OR of *d*_*PE*_>0 or *d*_*PS*_>0 denotes a benefit of the experimental treatment or the standard treatment over placebo, respectively. To assess whether *E* is superior to *S* (by at least a certain (pre-specified) relevant amount *Δ*≥0), we can estimate the probability

$$\upsilon = P(d_{PE} > d_{PS} + \Delta) $$

and base our decision on it. Under the consistency assumption, this equals to the definition

$$\upsilon = P(d_{ES} > \Delta) $$

and therefore, this index *υ* can also be applied in any Bayesian (pairwise or network) meta-analysis.

Of course, *Δ* can be chosen to be negative as well leading to a non-inferiority setting. Then, the probability of a treatment of being not less effective by more than a pre-specified amount compared to another treatment arm is estimated. In the following, it will be shown how the estimation of this probability can be realized.

### Estimation of *υ*

The log ORs are estimated via Bayesian methods. We assume that they are approximately normally distributed. As prior distributions, one can use (flat) normal distributions, resulting in a normal distribution as posterior. Let us assume that the posterior mean values of *d*_*PS*_ and *d*_*PE*_ are denoted by *μ*_*PS*,post_ and *μ*_*PE*,post_, respectively. One can then define a *Z* statistic as

$$Z = \frac{(d_{PE} - d_{PS} - \Delta) - (\mu_{PE, \text{post}} - \mu_{PS, \text{post}} - \Delta)}{\text{SE}(d_{PE} - d_{PS} - \Delta)}, $$

where

$$E(d_{PE} - d_{PS} - \Delta) = \mu_{PE, \text{post}} - \mu_{PS, \text{post}} - \Delta $$

and

$$\begin{array}{*{20}l} \text{SE}(d_{PE} - d_{PS} - \Delta) &= \text{SE}(d_{PE} - d_{PS})\\ &=\sqrt{\text{Var}(d_{PE} - d_{PS})} \end{array} $$

is the standard error of the difference of the log ORs. Thus, *Z* is asymptotically normally distributed as well.

Let *Φ*(·) denote the cumulative distribution function of the standard normal distribution. The probability of interest can then be approximated as

$$\begin{array}{*{20}l} &P(d_{PE} > d_{PS} + \Delta)\\ &\approx 1 - \Phi\left(\frac{-(\mu_{PE, \text{post}} - \mu_{PS, \text{post}} - \Delta)}{\sqrt{\text{Var}(d_{PE} - d_{PS} - \Delta)}}\right). \end{array} $$

It has to be noted that this approach is based on the approximation of the distribution of the log ORs by the normal distribution and is, therefore, only an approximation of *P*(*d*_*PE*_>*d*_*PS*_+*Δ*).

An estimate of this probability is then

$$\begin{array}{*{20}l} &\hat{P}(d_{PE} > d_{PS} + \Delta)\\ &=1 - \Phi\left(\frac{-(\hat{d}_{PE} - \hat{d}_{PS} - \Delta)}{\sqrt{\widehat{\text{Var}}(d_{PE} - d_{PS} - \Delta)}}\right), \end{array} $$

where $\hat {d}_{PE}$ and $\hat {d}_{PS}$ denote the estimates of the mean values of the posterior distribution of *d*_*PE*_ and *d*_*PS*_, respectively. The estimated posterior variance is denoted by $\widehat {\text {Var}}(d_{PE} - d_{PS} - \Delta) = \widehat {\text {Var}}(d_{PE} - d_{PS})$.

Estimation of this probability can be done within the MCMC approach in two different ways. The first approach is to estimate the (posterior) distributions of *d*_*PS*_−*d*_*PE*_−*Δ* directly. From this, we can estimate $\hat {d}_{PS} - \hat {d}_{PE} - \Delta $ as well as the variance $\widehat {\text {Var}}(d_{PE} - d_{PS} - \Delta)$. However, there is an even more intuitive way. In an MCMC estimation procedure, we store in every single iteration whether the parameter *d*_*PE*_ was larger than *d*_*PS*_+*Δ* or not. After the MCMC estimation is finished, we evaluate the relative frequency of runs where *d*_*PE*_>*d*_*PS*_+*Δ* within the MCMC approach to estimate the probability $\hat {P}(d_{PE} > d_{PS} + \Delta)$. An advantage of this approach is that it does not rely on the normal distribution and can therefore be applied in any NMA setting.

### Use of *υ* for Bayesian hypothesis testing

The index *υ* can be used to estimate the probability of superiority or non-inferiority between treatment arms with respect to the event probability. Therefore, it is a useful complement to the common results obtained in a NMA. Furthermore, this index can be used to make test decisions. Let us, again, assume that there are three treatment arms (*P*, *S* and *E*). Furthermore, we want to assess the following test problem:

$$\begin{array}{*{20}l} H_{0}: d_{PE} \leq d_{PS} + \Delta \quad \text{vs.} \quad H_{1}: d_{PE} > d_{PS} + \Delta, \end{array} $$

with *Δ*∈*R*. We can now use the index *υ* to perform a Bayesian hypothesis test in an NMA. If the value of *υ* exceeds a pre-specified value (for instance, 0.975, as an equivalent to a frequentist p-value of 0.025 which is typically used in a one-sided test procedure) we reject the null-hypothesis. Since the index *υ* is based on a Bayesian approach, it is unclear whether the test decisions coincide with the results of frequentist testing procedures. For this, a “probability matching prior” (PMP) has to be found as outlined, for example, in Datta and Sweeting [15]. We assume that the log ORs are normally distributed. It can be shown that in this case a uniform prior is a PMP [15]. In NMA, flat normal priors are commonly used which are very close to uniform priors if they are chosen sufficiently flat. However, since small deviations might still be present either because of the (flat) prior distribution or the approximation of the log OR via a normal distribution, we applied simulation studies to evaluate the characteristics of our approach.

### Some technical issues

As already discussed in the “Background” section, there are two ways to define an NMA model with a binary outcome. Either using the number of observations and the number of events per treatment arm assuming a binomial distribution, or using the approximately normally distributed log ORs.

In the next section, results from simulation studies will be provided where both approaches are compared. Therein, the method where the binomial distribution is used, is called *arm-based* approach. The method where ORs are modeled, is called *contrast-based* approach. The same distinction is done, for example, in the manual of the R package “netmeta” [16]. As a side note, the computation time of the contrast-based approach was substantially lower (in some situations about 40 times lower). Thus, from a computational point of view, this approach is much more efficient. From a technical point of view, the main difference between the arm-based and the contrast-based approach is that an additional level in the hierarchy of the Bayesian model is used. In the arm-based approach, a binomial distribution is estimated on the lower level, based on the number of successes (*y*_*ik*_) and the number of observations (*N*_*ik*_). On the upper level, the log ORs ($d_{A_{i}k}$) are estimated (model (1)). When using the trial-specific log ORs, there is only one level (model (2)).

Two different ways of estimating the probability *P*(*d*_*PE*_>*d*_*PS*_+*Δ*) have been presented above (note that this distinction is independent of the distinction between the contrast-based and the arm-based approach). The first option is to estimate the (posterior) distribution of *d*_*PE*_−*d*_*PS*_−*Δ* and the second one is to estimate *P*(*d*_*PE*_>*d*_*PS*_+*Δ*) directly during the MCMC procedure. In all simulation studies, both approaches were used in parallel. It became clear that the differences between the results where negligibly small. Thus, only the results from the second approach are presented, since it is the simplest way to estimate the index *υ*.

### Simulation study

Simulation studies were done to evaluate the testing approach. The main aim was to examine whether the approach maintains the type I error rate when used for hypothesis testing. For this, we have to define a cut-off value for a test decision. Analogously to a frequentist setting with a type I error rate of 0.025, we reject the null hypothesis *H*_0_: *d*_*PE*_≤*d*_*PS*_+*Δ* if $\hat {\upsilon } = \hat {P}(d_{PE} > d_{PS} + \Delta) \geq 0.975$.

A further issue was to examine the power of the approaches. Different settings regarding baseline risk, *d*_*PS*_, *d*_*PE*_, and *Δ* were used.

Binary data based on the assumption that the null hypothesis holds true were simulated and the rejection rate was estimated to examine the actual type I error rate. The boundary of the null hypothesis was considered, i.e., the data were simulated so that *d*_*PE*_=*d*_*PS*_+*Δ* holds true.

Three arms were compared (*P*: placebo; *S*: standard treatment; *E*: experimental treatment) in 16 studies, where four studies of each were simulated comparing *P* vs. *S*, *P* vs. *E*, and *S* vs. *E*, respectively, and another four studies were simulated including all three treatment arms. In each study, a sample size of 500 observations per treatment arm was used. We assume that the main interest was to compare the experimental treatment with the standard treatment. The success probabilities of the three arms were varied to examine the characteristics of our approach in different scenarios. The success probabilities of the placebo and the standard treatment arm were assumed to be equal which was done to simplify the simulation procedure; different values were chosen to evaluate different scenarios (*p*_*iP*_ = *p*_*iS*_ = 0.05, 0.1 or 0.2, *i* = 1, …,16). The success probability of the experimental arm was calculated such that *d*_*PE*_=*d*_*PS*_+*Δ* holds true. The values of *Δ* were chosen based on the ORs between the treatment arms. Eleven different ORs were used: log(1), log(1.05), log(1.1), log(1.2), log(1.5), log(2) (superiority), and log(1.05^−1^), log(1.1^−1^), log(1.2^−1^), log(1.5^−1^), log(2^−1^) (non-inferiority). The significance level was set to 0.025.

For each simulation scenarios, 50,000 iterations were used. Based on the results obtained in these scenarios, some further interesting data situations were examined. Firstly, a sample size of 1,000 observations per treatment arm with a success rate of 0.2 was used leading to a data situation where even approximate approaches should perform sufficiently well. Secondly, the sample size was lowered to 200 observations per treatment arm with a success rate of 0.1. The values for *Δ* were varied between log(0.9) and log(1.1) since the most often used values should be within this range. In a last scenario, extreme values of *Δ* were examined combined with a sample size of 400 observations per treatment arm using a success rate of 0.05.

We also evaluated our approach in situations where heterogeneity was present in the data. We used the same simulation settings as above (16 studies, 500 observations per arm). We did not vary *Δ* but set it to 0 thus considering a superiority setting. We simulated heterogeneity using the same values for *τ*^2^ as in Friede et al. [17]: 0.01, 0.02, 0.05, 0.1, 0.2, 0.5, 1, and 2. We, again, used three different baseline risk values: 0.05, 0.1, and 0.2. Random-effects models were fitted and 10,000 iterations per scenario were performed.

In a last step, we lowered the sample size per arm and trial to 50 patients, used a baseline risk of 0.1, and applied the same values for *τ*^2^ as before. Again, with 10,000 replications per scenario, random-effects models were fitted and evaluated.

Furthermore, the power of the testing approach was evaluated. Again, the main interest was to analyze the difference between the experimental and the standard treatment. The success rates in arm *P* and *S* were set to 0.1, assuming that *d*_*SE*_=1.15, and the sample size was varied from 100 to 1,000 observations per treatment arm. Per scenario, 10,000 iterations were used.

In all simulation scenarios, the consistency as well as the similarity assumption was assumed to hold true. For parameter estimation, MCMC techniques were used. Two chains with a burn-in of 20,000 followed by 40,000 runs with a thinning rate of 5 resulting in 8,000 samples per chain were generated to estimate the posterior distribution following Song et al. who used a similar setting [18]. The software R [19] in combination with JAGS (version 3.4.0 or higher, http://mcmc-jags.sourceforge.net/) and the R-packages rjags [20], doSNOW [21], foreach [22], coda [23], and iterators [24] were used to conduct the simulations. Since the computations were done on different systems and different work stations, different versions of the software packages were used. In the evaluation step, the package xtable [25] was used in addition.

### Illustrative example

To further illustrate the approach, we analyzed a real data example that was already evaluated elsewhere [6, 26]. The data are provided by the Smoking Cessation Guideline Panel [27].

In the data set, 24 trials comparing four different treatments about smoking cessation are included (A: “no contact”, B: “self-help”, C: “individual counseling”, and D: “group counseling”). The number of cessations and the number of observations are presented in Table 1. In the following, it is tested whether the treatment effects of arm *B*, *C*, and *D* are different from that of treatment arm *A* using a fixed-effect model. Here, the following three test problems for superiority (i.e., *Δ*=0) are assessed (no adjustment for multiple testing is performed):

$$\begin{array}{*{20}l} H_{0, 1}: d_{CA} \leq d_{DA}\quad &\text{vs.}\quad H_{1, 1}: d_{CA} > d_{DA}\\ H_{0, 2}: d_{CA} \leq d_{BA}\quad &\text{vs.}\quad H_{1, 2}: d_{CA} > d_{BA}\\ H_{0, 3}: d_{DA} \leq d_{BA}\quad &\text{vs.}\quad H_{1, 3}: d_{DA} > d_{BA} \end{array} $$

**Table 1:** Number of events and number of observations per trial for the illustrative data example (*y*_*ik*_ and *N*_*ik*_, *k*=*A*,*B*,*C*,*D*, respectively) [6, 26]

	A		B		C		D	
ID	*y*_*iA*_	*N*_*iA*_	*y*_*iB*_	*N*_*iB*_	*y*_*iC*_	*N*_*iC*_	*y*_*iD*_	*N*_*iD*_
1	9	140			23	140	10	138
2			11	78	12	85	29	170
3	79	702	77	694				
4	18	671	21	535				
5	8	116	19	146				
6	75	731			363	714		
7	2	106			9	205		
8	58	549			237	1561		
9	1	33			9	48		
10	3	100			31	98		
11	1	31			26	95		
12	6	39			17	77		
13	95	1107			134	1031		
14	15	187			35	504		
15	78	584			73	675		
16	69	1177			54	888		
17	64	642			107	761		
18	5	62			8	90		
19	20	234			34			
20	1	20					9	20
21			20	49	16	43		
22			7	66			32	127
23					12	76	20	74
24					9	55	3	26

It should be mentioned that these hypotheses were not pre-specified but the example is just presented to show the characteristics of our approach in a real data setting. Compared to the original data, the number of events was changed from 0 to 1 in two cases (study ID 9 and 20). This was done due to two reasons: If there are zero events in a treatment arm, an OR cannot be calculated. However, the contrast-based approach is based on ORs between treatment arms and thus the number of events had to be adjusted. As already mentioned above, the problem of rare events is common and discussed in the literature. In practice, a better choice may be to change the number of events from 0 to 0.5 and to add 0.5 to the number of observations [11]. However, the arm-based approach is based on a binomial distribution which is a discrete distribution. Thus, only integers can be used as numbers of events. Since a comparison of both approaches should be provided, the number of events was thus changed to 1.

An MCMC approach was implemented to estimate the parameters with 500,000 iterations after a burn-in of 100,000 iterations.

## Results

### Simulation study

In the following, we will present the simulation results. Due to convergence problems which resulted from zero counts, the results are sometimes based on slightly less than 50,000 or 10,000 runs, respectively. This is not mentioned in every single results description to improve readability.

**Fig. 1:**
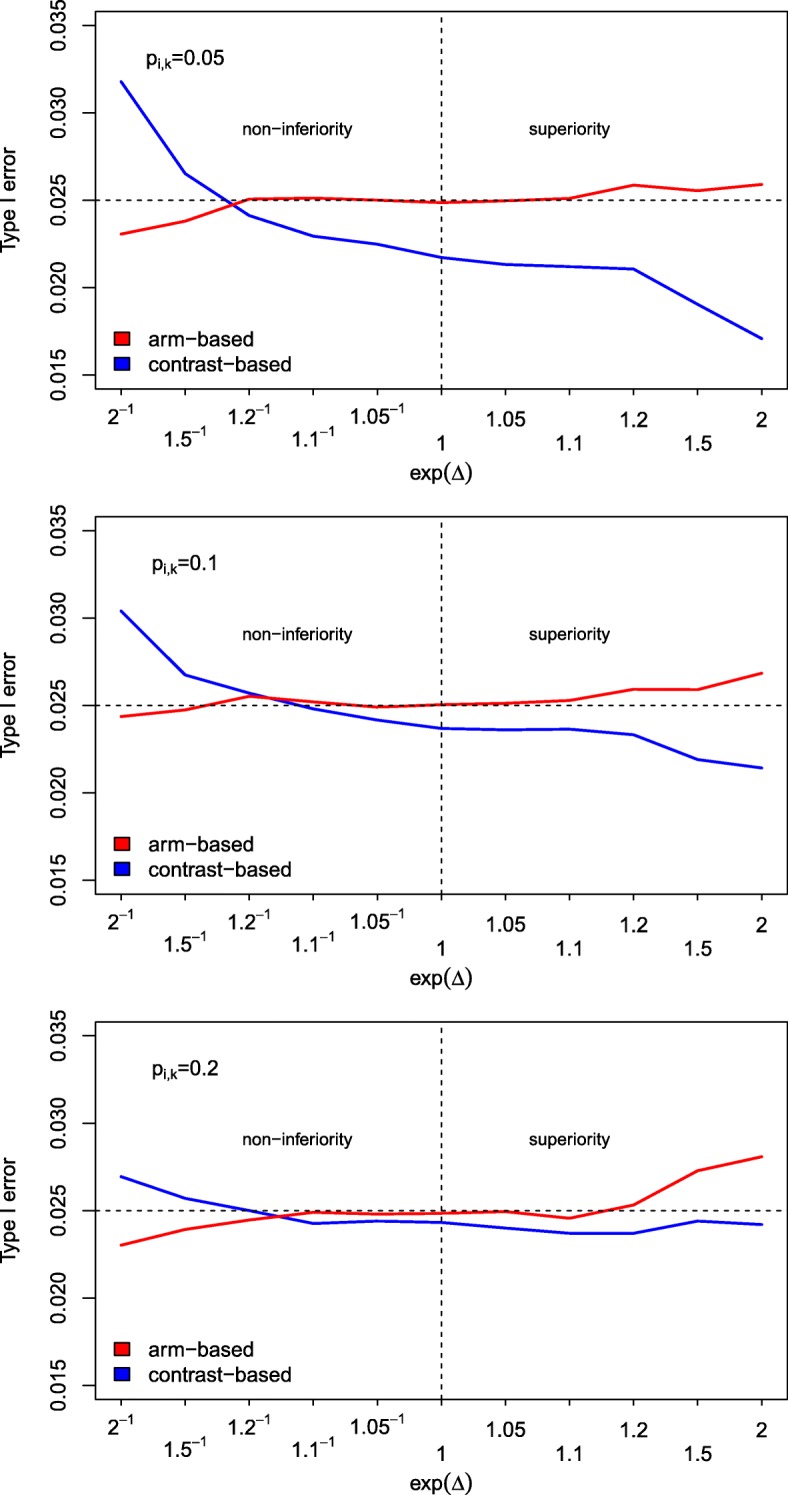
Simulated type I error rates. Simulated type I error rates for varying values of *Δ* (based on 50,000 runs). The sample size per treatment arm and the success rate were kept fixed at *N*_*ik*_=500 and *p*_*ik*_=0.05,0.1,0.2, respectively (*i*=1,…,16, *k*=*P*,*S*,*E*)

**Type I error rate:** The main interest was whether the approach maintains the type I error rate. In Fig. 1, the results of the first part of the simulation studies are shown. The number of observations per treatment arm was kept fixed (at 500 per treatment arm) and the value of *Δ* was varied, where three different success rates for treatment arms *P* and *S* were assumed (0.05, 0.1 and 0.2). The type I error rate using the contrast-based approach is close to the nominal level if the success rates are 0.1 or 0.2 and *Δ* is between log(1.2^−1^) and log(1.2) (Fig. 1). However, as soon as *Δ* is changed to more extreme values, it is slightly liberal in a non-inferiority setting (exp(*Δ*)<1) and slightly conservative in a superiority setting (exp(*Δ*)≥1). This characteristics is even more pronounced when the success rate is set to 0.05. Furthermore, one can see that the type I error rate tends to be higher the higher the success rate is. In contrast, the actual level of the arm-based approach is very close to the nominal one in most situations. Only if *Δ* and the success rate are relatively large, the type I error rates are slightly increased. If *Δ* is very small, the approach is slightly conservative. It is interesting to see that the lines in Fig. 1 cross. Thus, in some situations the arm-based and in some other situations the contrast-based approach is more conservative or liberal, respectively.

In the setting with 1,000 observations per treatment arm and study and values for *Δ* very close to 0, both approaches lead to very similar results. Both nearly maintain the type I error rate. The situations with 200 observations per treatment arm and *Δ*-values varying between log(0.9) and log(1.1) might be more interesting, since these values are more common in practice. In all these scenarios, the arm-based approach seems to perform slightly better than the contrast-based one, since it is less conservative but still maintains the type I error rate. Sometimes, the type I error rate was slightly above the nominal level. However, this exceedance can be regarded as negligible. In the last scenario, where extreme *Δ*-values were used, one can see that the contrast-based approach inflates the type I error rate in a non-inferiority setting while it is very conservative in the superiority trials. In contrast, the arm-based approach maintains the type I error rate in (even extreme) non-inferiority scenarios but inflates the type I error rate in a superiority setting. Table 2 summarizes these results.

**Table 2:** Simulated type I error rates of the testing approach in specific scenarios

*n*_*ik*_	*p*_*iP*_, *p*_*iS*_	*Δ*	contrast-based	arm-based
1000	0.2	log(0.9)	0.024	0.024
		log(1)	0.024	0.024
		log(1.1)	0.024	0.025
200	0.1	log(0.9)	0.023	0.025
		log(0.95)	0.022	0.025
		log(1)	0.022	0.026
		log(1.05)	0.021	0.025
		log(1.1)	0.023	0.026
400	0.05	log(0.5)	0.032	0.024
		log(2)	0.017	0.028

*n*_*ik*_ denotes the number of treatment arms, *p*_*iP*_ and *p*_*iS*_ the success rates in arm *P* and *S*, respectively, in trial *i* (*i*=1,…,16), and *Δ* is the non-inferiority or superiority margin, respectively. We used 50,000 simulated data sets to estimate the type I error rate. The nominal level of *α* was 0.025

**Fig. 2:**
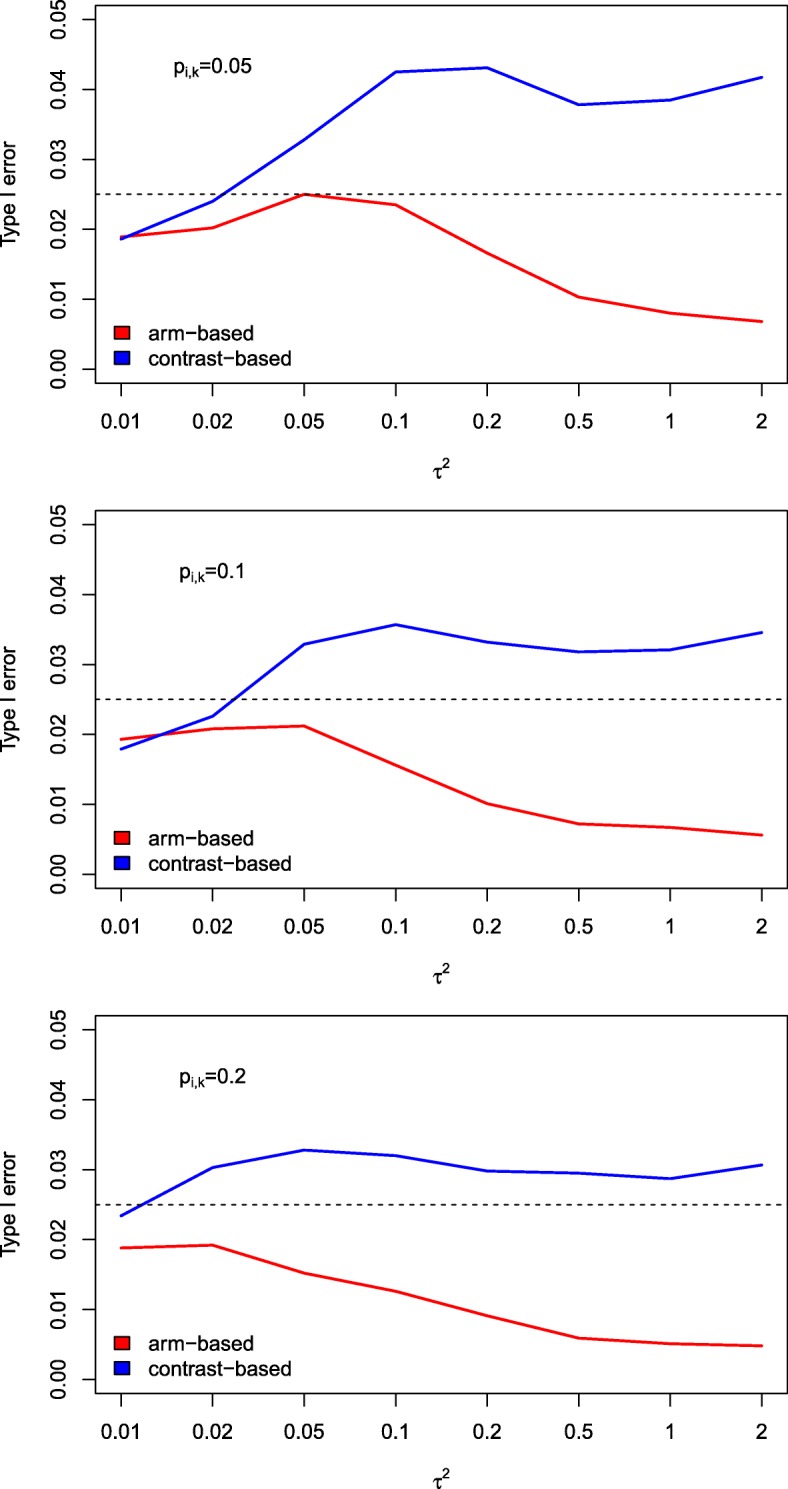
Simulated type I error rates (heterogeneity). Simulated type I error rates for varying values of *τ*^2^ (based on 10,000 runs). The sample size per treatment arm and the success rate were kept fixed at *N*_*ik*_=500 and *p*_*ik*_=0.05,0.1,0.2, respectively (*i*=1,…,16, *k*=*P*,*S*,*E*), while *Δ* was set to 0

When introducing heterogeneity, we saw that the results for the two approaches (arm-based and contrast-based) were more different. The arm-based approach always maintains the type I error rate but becomes very conservative in case of strong heterogeneity (see Fig. 2). The contrast-based approach, however, leads to slightly increased type I error rates for higher values of heterogeneity. Lowering the sample size to 50 patients per study did not, in general, lead to inflated type I error rates when the arm-based approach was used. Only in case of strong heterogeneity the type I error was slightly inflated, or the test behaved slightly too conservative in the situation of strong heterogeneity. In contrast, the effect-based approach led to an increased type I error rate in case of strong heterogeneity.

**Fig. 3:**
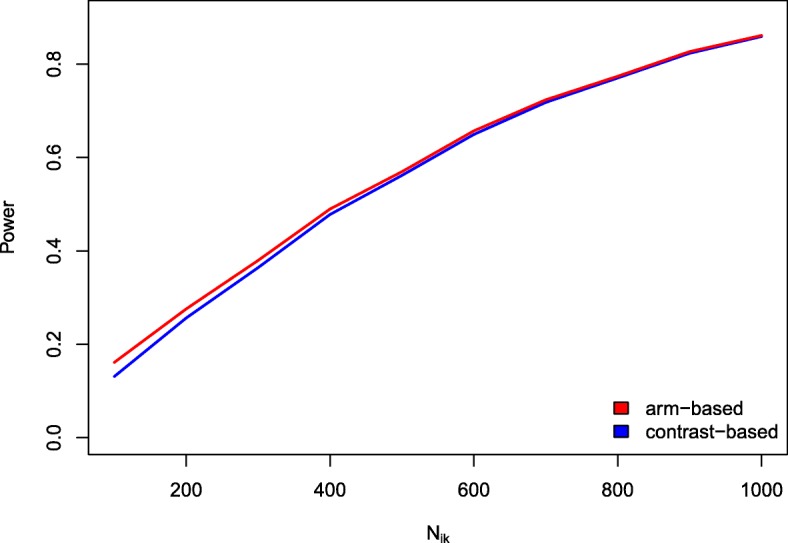
Power values. Power for the arm-based and contrast-based approach for a varying sample size *N*_*i*,*k*_ (based on 10,000 runs). The success rate was kept fixed at *p*_*ik*_=0.1 while the number of observations was varied (*i*=1,…,16;*k*=*P*;*S*;*E*). An OR of 1.15 was used for power simulation while *Δ*=0 was used

**Power** The investigations of the power showed that both approaches have a very similar performance. The arm-based approach resulted in slightly higher power compared to the contrast-based one (see Fig. 3). The difference decreased with increasing sample size. This was to be expected since the type I error rates of the arm-based approach were also slightly increased compared to the contrast-based one. However, one has to keep in mind that the arm-based method did not maintain the significance level in some situations and thus has to be used with care.

### Real data example

In Table 3, we provide the results for the data example. The estimated values for *υ* resulting from the arm-based and the contrast-based approach are presented for each pair of hypotheses. We can see that the arm-based approach always leads to a higher value of $\hat {\upsilon }$ than the contrast-based approach. If the cut-off for a test decision of 0.975 is applied, the following test decisions result. The first null hypothesis *H*_0,1_ cannot be rejected for both approaches. This means that the group counseling and the individual counseling are not significantly different. The second null hypothesis (*H*_0,2_) can be rejected according to both approaches that means that the individual counseling is significantly more effective than self-help. The third null hypothesis (*H*_0,3_) can be rejected with the arm-based but not when applying the contrast-based approach. Since we could see from our simulation study that the arm-based approach leads to type I error rates that are very close to the nominal level, the arm-based should be a proper choice. However, the safe (but maybe too conservative) option would be to apply the contrast-based approach and thus to maintain the null hypothesis in this case.

**Table 3:** Resulting values for $\hat {\upsilon }$ for the illustrative data example using the contrast-based and the arm-based approach

	Contrast-based	Arm-based
*H*_0,1_ vs. *H*_1,1_:	0.685	0.759
*H*_0,2_ vs. *H*_1,2_:	>0.999	>0.999
*H*_0,3_ vs. *H*_1,3_:	0.972	0.990

## Discussion

In this article, a method for hypothesis testing in an Bayesian NMA is presented. For this, an index was introduced that describes the probability of superiority or non-inferiority from a Bayesian perspective. We examined whether this index can also be used to make test decisions in a frequentistic sense. In a simulation study, two different approaches were compared, an arm-based and a contrast-based one. When there was no heterogeneity present in the data and fixed-effects models were applied, the observed type I error rates were very close to the nominal significance level while the arm-based approach led to slightly more favorable results in most situations. If the sample size is sufficiently high, both approaches maintain the type I error rate. If an extreme non-inferiority margin is used, only the arm-based approach led to valid results. An extremely large margin for relevant superiority, however, leads to an inflation of the type I error rate of the arm-based approach, and the contrast-based approach is then the better choice. However, in most situations in practice the deviations from the nominal type I error rate observed in our simulation studies are negligible. We also investigated the situation where heterogeneity is present in the data and saw that this can have a stronger impact on the type I error rate. However, even when the sample size was lowered to 50 patients per arm and trial, the type I error was still very close to the nominal level and only deviated slightly from it in case of strong heterogeneity. It is worth mentioning that our concept is not identical to a Bayesian posterior predictive p-value as described in Gelman et al. [28]. The index *υ* rather describes a Bayesian probability for superiority or non-inferiority.

There are some limitations of our simulation study. Of course, there are by far more data situations as those considered. However, we covered a range of common situations in medical research. There is also a lot of discussion about inconsistency in NMA models in the literature (see, for example, Dias et al. [29], or Krahn et al. [30]). In our simulation scenarios, it was assumed that there is no inconsistency present in the data which is a limitation of our study. Consistency is an assumption typically made in a standard NMA model but might be problematic in practice. In recent publications, this issue was addressed and solutions were proposed by applying more complex models [31–35]. However, in this work we focused on the standard NMA model. Note that when examining the type I error rate, the null hypothesis is assumed to hold true. Thus, the success rates in all treatment arms are exactly the same by design (or the same plus a pre-defined *Δ*) and therefore there is no inconsistency per definition.

A test decision can also be based on the 95% credible intervals around the point estimate of the log OR. If *Δ* is not included, the null hypothesis can be rejected. We compared this approach to the methods suggested in this article. The type I error rate tended to be slightly increased if the test decision was based on the credible interval compared to the approach based on *υ* but overall the results were very similar. Thus, it is not a considerable improvement compared to a test decision based on the credible intervals but rather a complement on the existing methodology.

## Conclusions

In conclusion, we proposed and discussed an index that can be used to test for superiority or non-inferiority of a treatment arm compared to another one within a Bayesian NMA. The estimation is done during the NMA model estimation and does not result in any (noticeable) additional computational cost. At the same time, the implementation is very easy. Obviously, this approach can also be applied in a straightforward way in any other data situation than binary data, as continuous data or a survival time, and is therefore a flexible tool.

However, as already mentioned, we did not cover all possible scenarios in our simulation study and, therefore, the index has to be used and interpreted with care. For example, as shown by Friede et al. [17] coverage of the credibility intervals decreases (and the type I error rate increases) substantially in case of rare diseases (low number of events), small populations, and strong heterogeneity. We did not discuss these situations here but it is clear that the same results for the index *υ* would have been observed as well. This shows that it is easy to generate examples that lead to invalid results. The choice of a proper prior distribution affects the results as well, as also described by Friede et al. [17]. Therefore, an adequate assessment of the data situation at hand has to be done before applying the approach discussed here or, in general, any NMA approach. It is hardly possible to define an approach that is valid and optimal for any situation in practice and we emphasize the limitations of the approach described in this paper.

## Abbreviations

NMA: Network meta-analysis
OR: Odds-ratio
